# A comparison of various lignin-extraction methods to enhance the accessibility and ease of enzymatic hydrolysis of the cellulosic component of steam-pretreated poplar

**DOI:** 10.1186/s13068-017-0846-5

**Published:** 2017-06-19

**Authors:** Dong Tian, Richard P. Chandra, Jin-Suk Lee, Canhui Lu, Jack N. Saddler

**Affiliations:** 1 Forest Products Biotechnology/Bioenergy Group, Department of Wood Science, Faculty of Forestry, University of British Columbia, 2424 Main Mall, Vancouver, BC V6T 1Z4 Canada; 2 State Key Laboratory of Polymer Materials Engineering, Polymer Research Institute of Sichuan University, Chengdu, 610065 China; 3 Clean Fuel Department, Korea Institute of Energy Research, Jeongeup, Jeonbuk 580-185 South Korea

**Keywords:** Steam pretreatment, Deep eutectic solvent, Enzymatic accessibility, Lignin extraction, Biorefinery

## Abstract

**Background:**

Current single-stage delignification-pretreatment technologies to overcome lignocellulosic biomass recalcitrance are usually achieved at the expense of compromising the recovery of the polysaccharide components, particularly the hemicellulose fraction. One way to enhance overall sugar recovery is to tailor an efficient two-stage pretreatment that can pre-extract the more labile hemicellulose component before subjecting the cellulose-rich residual material to a second-stage delignification process. Previous work had shown that a mild steam pretreatment could recover >65% of the hemicellulose from poplar while limiting the acid-catalysed condensation of lignin. This potentially allowed for subsequent lignin extraction using various lignin solvents to produce a more accessible cellulosic substrate.

**Results:**

A two-stage approach using steam and/or solvent pretreatment was assessed for its ability to separate hemicellulose and lignin from poplar wood chips while providing a cellulose-rich fraction that could be readily hydrolysed by cellulase enzymes. An initial steam-pretreatment stage was performed over a range of temperatures (160–200 °C) using an equivalent severity factor of 3.6. A higher steam temperature of 190 °C applied over a shorter residence time of 10 min effectively solubilized and recovered 75% of the hemicellulose while enhancing the ability of various solvents [deep eutectic solvent (DES), ethanol organosolv, soda/anthraquinone (soda/AQ) or a hydrotrope] to extract lignin in a second stage. When the second-stage treatments were compared, the mild DES treatment (lactic acid and betaine) at 130 °C, removed comparable amounts of lignin with higher selectivity than did the soda/AQ and organosolv pretreatments at 170 °C. However, the cellulose-rich substrates obtained after the second-stage organosolv and soda/AQ pretreatments showed the highest cellulose accessibility, as measured by the Simon’s staining technique. They were also the most susceptible to subsequent enzymatic hydrolysis.

**Conclusions:**

The second-stage pretreatments varied in their ability to solubilize and extract the lignin component of steam-pretreated poplar while enhancing the enzymatic hydrolysis of the resulting cellulose-rich residual fractions. Although DES extraction was more selective in extracting lignin from the steam-pretreated substrates, the organosolv and soda/AQ post treatments disrupted the cellulose structure to a greater extent while enhancing the ease of enzymatic hydrolysis.

**Graphical abstract:**
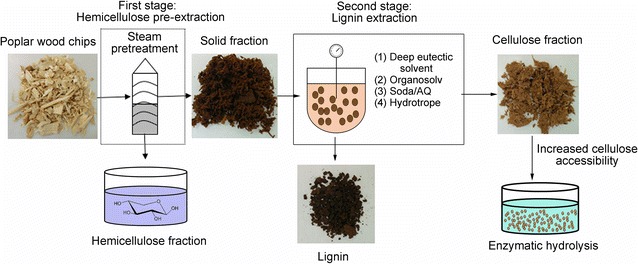
Effective hemicellulose removal via steam pretreatment followed by subsequent lignin extraction under acidic, alkaline or solvolytic conditions results in a highly accessible, more readily hydrolysed cellulose fraction.

## Background

An integrated biorefinery aims to use lignocellulosic biomass to produce multiple products including biofuels, platform chemicals and a wide range of material precursors similar to the array of products produced in contemporary oil/petroleum refineries [1, 2]. The key step to unlock an economically viable, integrated multi-product “biorefinery” is to effectively fractionate the cellulose, hemicellulose, lignin and extractives that comprise biomass into usable forms [3, 4]. However, the contrasting labile nature of hemicellulose and the recalcitrant nature of lignin limit the ability to fractionate these components using a single-stage process. Acidic conditions that solubilize hemicellulose can, in many cases condense the lignin, thereby decreasing its extractability and utility downstream [5]. Common single-stage delignification pretreatments such as ethanol organosolv, alkali and ionic liquids have been shown to be effective for extracting lignin. However, in many cases, this is at the expense of compromising the recovery of the hemicellulose component [6–12]. Thus, when these types of pretreatment strategies are employed to fractionate recalcitrant woody biomass, a higher loss of hemicellulose may occur due to the severe conditions required to solubilize the intractable lignin. Thus, the more labile nature of hemicellulose encourages the use of a two-stage-pretreatment approach, where the hemicellulose component is initially solubilized and recovered at mild conditions prior to the extraction of lignin.

In a previous work, it was shown that an initial mild steam-pretreatment stage could recover >65% of the hemicellulose from poplar, while facilitating subsequent lignin extraction by an acidic organosolv treatment [13]. This earlier steam-pretreatment work was carried out at a low severity [Eq. (1) as determined by the pretreatment temperature and residence time], to provide effective recovery of the hemicellulose sugars, while enhancing enzymatic hydrolysis of the residual, water-insoluble cellulose [13, 14].

In other work, ethanol organosolv pretreatment has been shown to be an effective method for extracting lignin from steam-pretreated poplar during a second-stage of pretreatment [15]. However, the acidic conditions that are typically utilized during the ethanol organosolv process have been shown to limit lignin selectivity due to the acidic hydrolysis of the cellulose component [7, 16]. Similarly, the acidic conditions that catalyse lignin depolymerization during organosolv are accompanied by competing condensation reactions that can also limit lignin removal [17]. Thus, one of the goals of the present work was to compare ethanol organosolv pretreatment to other delignification methods such as deep eutectic solvents (DESs), soda/anthraquinone (soda/AQ) and hydrotropic treatments. As these alternative delignification methods are performed under either mildly acidic or alkaline conditions, we wanted to determine if these solvent systems might extract lignin with greater selectivity. As DESs are formulated using a combination of hydrogen-bond donors and acceptors, they are considered to be anon-volatile way of dissolving lignin [18–20]. In contrast, the soda/AQ method is a commercialized pulping process that delignifies through the alkaline cleavage of lignin ether bonds and is thought to be less prone to competing condensation reactions compared to acidic pretreatments [21, 22]. The third delignification method that was evaluated used hydrotropic salts which are water-soluble organic amphiphilic compounds that form molecular aggregates when the salt concentration is sufficiently high. Hydrotropic salts have been shown to dissolve large amounts of lignin [23, 24]. However, it should be noted that each of these delignification methods limits the effective recovery of the hemicellulose component from the spent liquors [11, 25] and can result in some hemicellulose degradation [7].

In the present work, a consistent steam-pretreatment severity of 3.6 was obtained, by varying the temperature and residence time, to determine the conditions that maximized hemicellulose recovery. The resulting steam-pretreated “hemicellulose-poor” poplar wood was then subjected to delignification using either soda/AQ, hydrotropic salts, or DES formulated using lactic acid and betaine. These delignification methods were compared to ethanol organosolv pretreatment to determine if they could selectively extract a greater amount of lignin from the steam-pretreated poplar biomass. The four solvent-insoluble cellulose fractions were further characterized to determine how the cellulosic component might have been altered by the different delignification approaches while, hopefully, enhancing their susceptibility to enzymatic hydrolysis. It was apparent that the delignification approaches varied greatly in their ability to selectively remove lignin from the steam-pretreated substrate, while producing a cellulose-rich fraction that was readily hydrolysed by cellulases.

## Experimental

### Materials

Poplar wood chips (particle size, about 0.5–3 cm) were supplied by the BC Ministry of Forests, Canada. Kraft lignin (Product No. 370959), lactic acid and betaine were purchased from Sigma-Aldrich. Cellic CTec3 cellulase was supplied by Novozymes (Davis California). Direct orange (Pontamine Fast Orange 6RN, Lot No. 814071) dye was supplied by Pylam Products Co. Inc. (Garden City, NY).

### Steam pretreatment of poplar wood

Steam pretreatment of poplar was carried out using equipment and under the general conditions as described previously [13]. A severity factor of log*R*_0_ = 3.6 was used for all of the treatments meaning that the residence time was decreased as the temperature was raised over a range of 160–200 °C (Table 1), The pretreatment severity (*R*_0_) can be calculated using the following equation [13]:

1 $$R_{0} = t \cdot \exp \left[ {\left( {\frac{T - 100}{14.75}} \right)} \right],$$

where *t* is residence time in minutes and *T* is pretreatment temperature in °C. Prior to steam pretreatment, 200 g of poplar wood chips was placed in plastic bags and impregnated with 0.7 wt% (based on dry wood) dilute sulphuric acid solution at a solid:liquid ratio of 1:1 and stored at room temperature overnight. The impregnated biomass samples were subsequently loaded into a 2-L Stake Tech II steam gun [Stake Tech II batch reactor, SunOpta (formerly Stake Technologies) of Norval, ON, Canada]. After steam pretreatment, the resulting slurry was collected, and the water-soluble fraction was separated from the solid fraction by vacuum filtration. The solid fraction was washed with 1 L water and stored at 4 °C for further use. The liquid fraction, including the washing liquor was collected and centrifuged.

**Table 1:** Steam-pretreatment conditions at a constant severity of 3.6 obtained by manipulating temperatures and residence times

Sample	Temperature (°C)	Time (min)	H_2_SO_4_ loading (%)^a^	Severity (*R*_0_)
SP1	200	5.0	0.7	3.6
SP2	190	9.9	0.7	3.6
SP2^#^	190	9.9	0.0	3.6
SP3	180	19.4	0.7	3.6
SP4	170	38.2	0.7	3.6
SP5	160	75.3	0.7	3.6

^a^ Based on the weight of dry poplar wood chips

^#^ refers to the sample steam pretreated at 190 °C without the addition of H_2_SO_4_

### Lignin extraction

Lignin-extraction methods using DES, soda/AQ, hydrotrope and organosolv were compared. In an attempt to optimize DES extraction, the molar ratio of lactic acid to betaine was assessed based on its lignin dissolution capacity, thermal stability and acidity in water. Defined amounts of lactic acid and betaine were stirred at 80 °C until a homogeneous colourless DES liquid was formed. The thermal stability of the resulting DES was determined by measuring the weight loss of the mixture after heating at 130 °C for 12 h. The solubility of Kraft lignin in DES was determined by the cloud point method [19] where vials containing 2 g of DES were first placed on a hot plate at constant temperature of 80 °C and Kraft lignin was added in portions of about 0.5 wt% of DES each time under vigorous stirring. Additional lignin was continuously introduced until the solution became turbid. The cloud point was recorded if the sample did not become clear after equilibration for 3 h. The acidity of the DES was measured using a pH meter (Fisher Scientific, model XL20, Canada).

The effectiveness of DES extraction of lignin was assessed at atmospheric pressure on a hot plate equipped with a digital controller and magnetic stirring. One gram of poplar biomass (dry matter) and 20 g of DES were transferred to a 100-mL conical flask and heated at 130 °C for 3 h under continuous stirring. The reaction mixture was then cooled to about 80 °C, after which 30 mL of acetone/water mixture (50/50 by volume) was added to fractionate the DES and lignin from the cellulose-rich pulp. The pulp was subsequently washed twice using an acetone/water mixture with a subsequent two hot-water washing steps to remove traces of the DES and lignin.

Lignin extractions using organosolv, soda/AQ and hydrotropic treatments were performed according to conditions used in previous works (Table 2) [13, 21, 24, 26]. In brief, 100 g (dry weight) of steam-pretreated poplar biomass was heated at 170 °C for 1 h at a liquid–solid ratio of 7:1 using a four-vessel (2 L each) rotating digester (Aurora Products, Savona, BC, Canada). At the end of each pretreatment run, the vessels were cooled to room temperature in a water bath. The solid substrates were subsequently filtered using a Whatman No. 1 paper and thoroughly washed prior to further characterization.

**Table 2:** Conditions for lignin extraction used for second-stage pretreatments (170 °C, 1 h, liquid:solid = 7:1 by v/w)

Methods	Extraction solvent	Catalyst	Washing procedure	Lignin precipitation	Ref.
Organosolv	Ethanol/water 50/50 by wt	1% H_2_SO_4_	Ethanol/water, 50/50 by wt then hot water	10× volume of hot water	[15]
Soda-AQ	14 wt% active alkali in water	0.1% AQ	Hot water	Adjusting pH to 2.0	[23]
Hydrotrope	30 wt% sodium salicylate in water	0.17% formic acid	Hot water	10× volume of hot water	[26]

### Characterization of the cellulose-rich fractions after two-stage pretreatments

The chemical composition of the substrates was determined using the Klason procedure (TAPPI Standard Method T-222) [27]. In brief, 0.7 mL of 72% H_2_SO_4_ was added to 15 mL of the liquid samples, and the volume was adjusted to 20 mL with water. The samples were then autoclaved at 121 °C for 1 h prior to analysis. The monosaccharides were determined using a DX-3000 high-performance liquid chromatography (HPLC) system (Dionex, Sunnyvale, CA), equipped with an anion exchange column (Dionex CarboPac PA1), and fucose as the internal standard. The column was eluted with deionized water at a flow rate of 1 mL min^−1^. Aliquots (20 μL) were injected after being passed through a 0.45-µm nylon syringe filter (Chromatographic Specialties Inc., Brockville, ON, Canada). The baseline stability and detector sensitivity were optimized by the post-column addition of 0.2 M NaOH at a flow rate of 0.5 mL min^−1^ using a Dionex AXP pump. The column was re-conditioned using 1 M NaOH after each analysis.

The X-ray diffraction patterns (XRD) of raw and-pretreated poplar substrates were determined using a Bruker D8-Advance powder X-ray diffractometer and Cu-Ka radiation (*k* = 0.1540 nm) at an accelerating voltage of 40 kV and a current of 40 mA. The data were collected for 2*θ* = 5°–80° with a step interval of 0.04°. The crystallinity index (% CrI) was calculated using the Segal method [28]:

2 $${\text{CrI}}\left( \% \right) = \frac{{I_{002} - I_{\text{am}} }}{{I_{002} }}\; \times \;100,$$

where *I*_002_ is the maximum intensity of approximately 22.7° and *I*_am_ corresponds to the minimum intensity located at 2*θ* close to 18°. The average crystal size *τ* was determined by the Scherrer equation [29]:

3 $$\tau = \frac{K\lambda }{\beta \cos \theta },$$

where *K* is a constant that depends on the crystal shape (1.0 in this case), *λ* is the wavelength of the incident beam in the diffraction experiment, *β* is the full width at half maximum in radians and *θ* is the position of the peak (half of the plotted 2*θ* value).

The average degree of polymerization (DP) was calculated based on the intrinsic viscosity value [30]:

4 $${\text{DP}}^{0.905} = 0.75\left[ \eta \right],$$

where [*η*] is intrinsic viscosity in cm^3^ g^−1^ determined by the viscosity (25 °C) of a cellulose solution in cupriethylenediamine (CED) solution using Ubbelohde viscometer according to ASTM D1795. Prior to dissolution in CED, the substrate was treated with sodium chlorite followed by a sodium hydroxide solution treatment to remove residual lignin and hemicellulose, respectively, according to our previous study [31].

Cellulose accessibility was determined using the Simon’s staining (SS) technique according to the modified procedure proposed by Chandra et al. [32]. Water retention values (WRVs) were determined and calculated according to TAPPI Useful Method-256.

### Enzymatic hydrolysis

Enzymatic hydrolysis was carried out at either 2 or 10% (w/v) solid loading in sodium acetate buffer (50 mM, pH 4.8), 50 °C, 150 rpm in a benchtop orbital shaker (MaxQ 4000, Barnstead/Lab-Line) with an enzyme loading (Cellic CTec3, protein content, 234 mg mL^−1^) of 8 mg _enzyme_ ${\text{g}}_{\text{glucan}}^{ - 1}$ cellulose. 0.5 mL of each hydrolysate was taken at certain time points and incubated on a hot plate at 100 °C for 10 min to deactivate the enzyme. The sample was subsequently centrifuged, and the supernatant collected and analysed for sugar release using HPLC.

## Results and discussion

### Steam pretreatment at a constant severity to achieve hemicellulose extraction and recovery prior to lignin removal

**Fig. 1:**
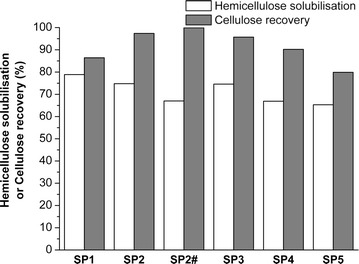
Hemicellulose solubilization and cellulose recovery after steam pretreatments performed at a constant severity of 3.6 with the addition of 0.7 wt% H_2_SO_4_ (on biomass) and temperatures increasing in 10 °C increments from 160 to 200 °C denoted, SP1–SP5 respectively. Sample SP2# was treated without the addition of the H_2_SO_4_ catalyst

Earlier work has shown that low-severity steam pretreatment could result in good hemicellulose recovery and solubilization [13] and that specific severity factor could be achieved using either a shorter residence time at higher temperatures or by reducing the temperature and extending the residence time [33]. Although previous work has shown that steam pretreatment of poplar at a temperature of 200 °C and a residence time of 5 min (severity factor, *R*_0_ of 3.6) was effective in solubilizing hemicellulose while preserving the cellulose component [14], hemicellulose recovery has never been optimized. Therefore, in an attempt to maximize hemicellulose recovery, several steam-pretreatment conditions were compared, using a constant severity of 3.6, at temperatures ranging from 160 to 200 °C and residence times ranging from 75.3 to 5 min (Table 1) [13, 27]. A 0.7 wt% H_2_SO_4_ loading based on the dry poplar wood was added to improve hemicellulose solubilization/recovery at the lower pretreatment temperatures/shorter residence times [34]. Although each of the steam pretreatments conditions resulted in some hemicellulose solubilization (Fig. 1), increasing the temperature from 160 to 200 °C while decreasing the residence time from 75.3 to 5.0 min gradually enhanced hemicellulose solubilization from 43.2 to 55.0%. The addition of 0.7 wt% of H_2_SO_4_ as a catalyst resulted in improvement of 9.1% in hemicellulose recovery (Sample SP2) compared with the sample that was treated without the H_2_SO_4_ catalyst (SP2#). This was likely due to the enhanced hemicellulose solubilization catalysed by the additional acid [27]. However, it was also apparent that increasing the temperature or extending the residence time also compromised the recovery of the cellulose component (Sample SP1 and SP5, Fig. 1). This indicated that, although the severity equation is the widely accepted method to describe the intensity of a given steam-pretreatment condition, changing the temperatures and residence times over a relatively wide range at a constant severity factor indicates the limitation of using just the severity equation to estimate the likely effectiveness of pretreatment. It was also apparent that, at least for the case when using poplar, the temperature of pretreatment was the most influential determinant of treatment severity. As the pretreatment with supplemented sulphuric acid addition, at a temperature of 190 °C and a residence time of 9.9 min resulted in the best compromise between hemicellulose solubilization/recovery and cellulose recovery, these conditions, (SP2) were used to produce the steam-pretreated biomass for the subsequent delignification treatments (Fig. 1). Prior to comparing the delignification treatments, the steam-pretreated substrates were subjected to enzymatic hydrolysis to provide a “base-case” of their ease of enzymatic hydrolysis without delignification.

**Fig. 2:**
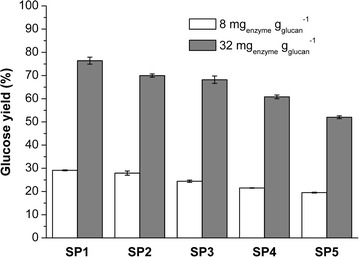
Glucose yield of sample SP1–SP5 after enzymatic hydrolysis at low (8 mg _enzyme_ ${\text{g}}_{\text{glucan}}^{ - 1}$) and high (32 mg _enzyme_ ${\text{g}}_{\text{glucan}}^{ - 1}$) enzyme loadings

As indicated in an earlier work [35], the residual lignin present in steam-pretreated woody substrates (>30%) proved to be problematic for the subsequent enzymatic hydrolysis of the cellulose, despite the use of a relatively high enzyme loading of 32 mg enzyme per ${\text{g}}_{\text{glucan}}^{ - 1}$. Without delignification, even at a low solids loading of 2% (w/v), only 70% cellulose hydrolysis was achieved (Fig. 2). As mentioned earlier, lignin condensation can occur under acidic pretreatment conditions. This can result in an increase in the molecular weight of the lignin, compromising its downstream extractability. In order to determine the effects of steam pretreatment on the ease of lignin extraction, four delignification methods were applied to the SP2 substrate. Although previously determined conditions could be used for the soda/AQ, hydrotrope and organosolv treatments (Table 2), there have been only limited reports of the application of DESs to woody biomass substrates [36]. Therefore, prior to assessing all the four delignification methods, we first wanted to identify optimal conditions that would maximize the dissolution of lignin using a betaine and lactic acid DES that had been shown to have potential for lignin dissolution [19].

### Lignin extraction using DES

Previous work has shown that lactic acid and betaine can both be produced from natural resources and that their combination can effectively dissolve lignin [19]. Initially, we compared the molar ratios of the lactic acid and betaine and the mixtures ability to dissolve Kraft lignin while monitoring the pH and thermostability of the DES mixture. When the molar ratio of lactic acid to betaine exceeded 2.5, the resulting DES was able to dissolve more than 23 wt% of the Kraft lignin at 80 °C (data not shown), which was as good as some of the previous results obtained using ionic liquids [37, 38]. When the molar ratio of the lactic acid to betaine was increased, this enhanced lignin dissolution and acidity. However, raising the ratio of the lactic acid also compromised the thermal stability of the resulting DES and increased the volatility of the mixture [39]. Since the DES with a molar ratio of 2.5 was able to dissolve Kraft lignin while exhibiting considerable thermal stability, this mixture was used for the subsequent lignin extractions applied to the SP2 biomass.

**Fig. 3:**
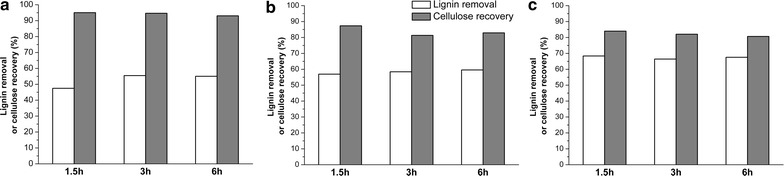
Lignin removal and cellulose recovery resulting from deep eutectic solvent (DES) extraction of steam-pretreated poplar (SP2). Poplar wood was steam pretreated at 190 °C for 9.9 (SP2) and then subjected to DES extraction at varying temperatures of **a** 130 °C, **b** 160 °C, **c** 190 °C and residence times of 1.5–6 h

It was apparent that varying the DES extraction conditions, such as the residence time (1.5, 3, 6 h) and extraction temperature (130, 160, 190 °C), significantly influenced lignin removal and cellulose recovery (Fig. 3). The amount of lignin extracted from SP2 ranged from 47.5 to 68.4% with a corresponding cellulose recovery of 95.0 to 80.7%. However, the enhanced lignin removal, which resulted from the increasing reaction temperature, was achieved at the expense of reduced cellulose recovery (Fig. 3). It was also evident that the increasing residence time had only a marginal effect on lignin removal. This result was quite different from a typical ionic liquid-extraction process where extending the residence time typically results in an increase in lignin removal [11]. A possible explanation for this observation is that, in a typical ionic liquid extraction process, the entire biomass sample is dissolved with subsequent precipitation in an anti-solvent [40, 41]. Therefore, the extended residence time enhances the dissolution of the complex cell wall structure of the biomass in the ionic liquid. However, during DES extraction, the lignin is selectively solubilized from the biomass and subsequently separated at the interface between the wood fibres and the DES. Thus, the solubilized “DES-lignin” could be precipitated in an anti-solvent to separate it from the residual biomass. In all subsequent DES extractions, a temperature of 130 °C and a residence time of 3 h was used.

### Evaluation of the second-stage targeted lignin-extraction processes

When the chemical composition of the resulting two-stage-pretreated substrates were compared (Table 3), although all of the second-stage extraction methods could effectively delignify the SP2 substrate, they varied considerably in their ability to selectively solubilize lignin without affecting the carbohydrate component (Table 3). When the selectivity of a given delignification process was estimated by the dividing the solids yield (“pulp yield”) by the lignin content of the resulting substrate, it was apparent that the hydrotropic extraction had a selectivity of 4.4. This indicated that the lignin content was higher compared with the other extraction methods (19%), while the solids yield was approximately 85%. Although hydrotropic delignification appeared to be highly specific, it removed less lignin from the SP2 substrate compared with the other delignification methods. Despite their differences in approach (acid vs alkaline), the soda/AQ and ethanol organosolv pretreatments resulted in similar solids yields and lignin contents (Table 3). This could be a result of similarities between these two lignin-removal approaches, as both processes are thought to proceed through a “quinone-methide” type intermediate initiated by the scission of the α-O-4 lignin substituent [42, 43].

**Table 3:** Solid yield, delignification and chemical composition of the four cellulose-rich fractions resulting from various two-stage pretreatments

Sample	Solid yield (%)^a^	Delignification		Chemical composition (%)					
		Extent (%)^b^	Selectivity	Glu	Xyl	Ara	Gal	Man	Lignin
Raw poplar	–	–	–	48.9	14.6	0.4	0.6	2.9	27.5
SP2	–	–	–	63.6	4.9	0.0	0.1	1.2	27.4
DES-SP2	82.6	52.4	5.2	72.9	4.3	0.0	0.0	0.0	15.8
Organosolv-SP2	66.3	63.2	4.4	75.1	2.2	0.0	0.0	2.4	15.2
Soda/AQ-SP2	72.5	54.5	4.2	70.5	2.3	0.0	0.0	2.4	17.2
Hydrotrope-SP2	84.5	40.5	4.4	69.5	3.3	0.0	0.0	2.3	19.3

–, cannot be determined

^a^ Based on the dry weight of the substrate resulted from steam pretreatment at 190 °C for 9.9 min (SP2)

^b^ Based on the lignin content in SP2 substrate

It was apparent that the DES method, with a selectivity of 5.2, was the most effective in selectively solubilizing the lignin while preserving the carbohydrate component. Compared with ethanol organosolv extraction, this resulted in the recovery of 66% of the solid substrate and a lignin content of 15%, the DES treatment of the SP2 substrate resulted in a solids yield of 83% and a lignin content of 16% (Table 3). It is likely that the acid-catalysed steam pretreatment (SP2) of poplar resulted in a decrease in the degree of polymerization of the cellulose and hemicellulose. This consequently increased the susceptibility of these carbohydrates to acid hydrolysis/solubilization and chain cleavage/peeling reactions during the subsequent organosolv and alkaline soda/AQ treatments respectively [16, 44]. The milder conditions employed during the DES treatments likely aided in the retention of the cellulose component in the solid substrate fraction while allowing for the selective removal of lignin. The variations in lignin selectivity between the delignification methods likely affected the characteristics and accessibility of the cellulose. A decrease in selectivity probably indicated that the solvent system used for delignification may have also reacted with carbohydrate component of the steam-pretreated biomass (SP2). For example, it has been shown that acidic ethanol organosolv pretreatments decrease the molecular weight of cellulose to a greater extent than do soda/AQ or DES treatments [45]. To see if the different treatments influenced substrate characteristics such as cellulose accessibility, degree of polymerization, crystallinity, etc., we next assessed the susceptibility of each substrate to enzymatic hydrolysis and compared this profile with the various substrate characteristics.

### Characterization of cellulose-rich fractions obtained from two-stage pretreatments

The cellulose-rich substrates from the two-stage treatments were characterized for cellulose crystallinity, degree of polymerization, and accessibility using the WRV and direct orange dye adsorption assays. These measurements were performed to try to determine how the physical/chemical properties of the substrate might influence the ease of enzymatic hydrolysis of the cellulose component. The ease of hydrolysis of a pretreated biomass substrate has been shown to be affected by cellulose accessibility and by characteristics such as the crystallinity and degree of polymerization of the cellulose [46]. At the fibre level, the overall porosity/accessibility of pretreated lignocellulosic substrates has been estimated by methods such as Simon’s staining and WRV which have also provided effective estimates of a given substrate’s susceptibility to enzymatic hydrolysis [27].

The increase in crystallinity of the single-stage and two-stage-pretreated poplar from 52 to 64% after a mild steam pretreatment (SP2) was likely due to the removal of amorphous hemicellulose from the residual substrate (Table 4) [47]. The partial removal of the amorphous lignin and hemicellulose in the second-stage pretreatments also resulted in a further increase in the CrI values (Table 4) [47]. A transition to larger crystallites was particularly evident for the substrates that were subjected to the two-stage-pretreatment regimes, with the exception of the steam-DES-treated substrates.

**Table 4:** Segal crystallinity index (CrI), crystal size, degree of polymerization (DP), water retention value (WRV) and direct orange dye adsorption of raw poplar, steam-pretreated poplar and cellulose-rich pulp from the four two-stage pretreatments

Sample	Segal CrI (%)	Crystal size (nm)	DP	WRV (%)	Adsorption of direct orange (mg g^−1^)
Raw poplar	51.5	1.8	–	–	–
SP2	63.7	2.7	622	177	47.6
DES-SP2	65.1	2.9	615	226	71.4
Organosolv-SP2	74.2	3.8	212	262	72.5
Soda/AQ-SP2	68.6	3.5	479	207	81.9
Hydrotrope-SP2	71.0	2.9	523	200	63.6

–, cannot be determined

It was apparent that the DES pretreatment preserved the amorphous cellulose in the substrate since it exhibited a smaller crystal size compared with the organosolv=, soda/AQ= and hydrotrope-pretreated substrates (Table 4). It appeared that DES primarily extracted the lignin component without disrupting the cellulose structure. In contrast, the other three chemical extractions likely solubilized the hemicellulose and amorphous regions of cellulose, consequently increasing the residual substrates’ overall crystallinity. After mild steam pretreatment of poplar wood, the DP of the substrate decreased to 622 (Table 4). However, subsequent DES extraction of the steam-pretreated poplar (SP2) had only a limited effect on the DP, indicating the high specificity of the DES system for lignin extraction. In contrast, organosolv pretreatment resulted in a significant reduction in DP to 212 (Table 4), likely due to hydrolysis undergone by cellulose in the acidic conditions applied during the organosolv process. The DP has been shown to play a role in cellulose accessibility to enzymes [48].

As discussed earlier, overall substrate accessibility is also governed by macroscopic properties such as fibre characteristics (size, porosity coarseness, etc.) as well as the amount and distribution of hemicellulose and lignin. Earlier work has used a modified Simon’s staining method and the adsorption of direct orange dye in particular to estimate the area of pretreated biomass that is likely accessible to cellulase enzymes [32]. In a complementary fashion, the WRV provides an estimate the amount of water that can be retained by the inner pores of a given pretreated substrate [49]. The WRVs are also influenced by the hydrophilicity of a given pore, as a porous substrate that is rich in hydrophobic lignin may result in a lower water retention value. Both the WRV and Simon’s stain values of the cellulose-rich substrates increased, compared with the original steam-pretreated substrate (SP2), indicating enhanced overall cellulose accessibility after the various second-stage pretreatments that removed lignin (Table 4). The organosolv- and soda/AQ-extracted substrates absorbed the most direct orange dye, indicating enhanced accessibility (Table 4). The organosolv-pretreated substrate also had the highest WRV, likely due to the decrease in DP exposing a greater amount of cellulose chain that enhanced water retention [48]. It was apparent that, compared with the hydrotropic treatment where the lignin fragmentation mechanism occurs via solubilization rather than fragmentation, organosolv, soda/AQ and DES second-stage pretreatments resulted in the greatest increase in cellulose accessibility as determined by both the WRV and Simon’s staining methods.

### Enzymatic hydrolysis of the cellulose-rich fractions

**Fig. 4:**
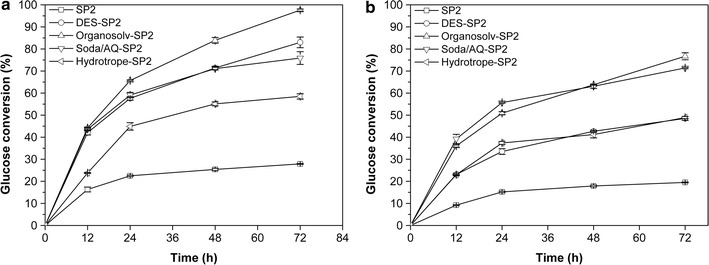
Enzymatic hydrolysis of the cellulose-rich substrates from the two-stage pretreatments of poplar **a** 2% w/v and **b** 10% w/v solids loading with an enzyme loading of 8 mg _enzyme_ ${\text{g}}_{\text{glucan}}^{ - 1}$

The four, two-stage-pretreated substrates and the SP2 control (single-stage pretreatment), were hydrolysed at an enzyme protein loading of 8 mg _enzyme_ ${\text{g}}_{\text{glucan}}^{ - 1}$ at both 2 and 10% (w/v) solids loadings for 72 h (Fig. 4). At the 2% solids loading, the highest hydrolysis yield (97.6%) was observed on the organosolv-SP2 substrate, followed by DES-SP2 (83.0%), soda/AQ-SP2 (75.9%) and hydrotrope-SP2 (58.5%). These hydrolysis yields were all considerably higher than the 27.9% obtained after hydrolysis of SP2 (Fig. 4a). The improved hydrolysis yields obtained after partial lignin removal in the second stage seemed to confirm earlier observations that the lignin retained in the substrate after steam pretreatment limited the enzyme accessibility to the cellulose component [35]. When the solids loading was increased from 2 to 10%, the DES substrate in particular underwent the most pronounced decrease in hydrolysis yields (Fig. 4b). As discussed earlier, DES is highly specific in removing lignin compared with the soda/AQ and the organosolv treatments (Table 3). This high level of specificity likely resulted in the higher degree of polymerization of the DES-derived cellulose which likely contributed to the observed reduction in hydrolysis yields at higher solids loading (Table 4). At a higher solids loading, the endo- and exoglucanases have been shown to be jointly responsible for deconstructing the biomass substrate [50]. The higher DP value of the DES substrate results in fewer chain ends being available to the exoglucanases, thus slowing the rate of biomass deconstruction at higher solids loading. In contrast, the significant reduction in cellulose DP after the organosolv-SP2 (76.7%) and soda/AQ-SP2 (71.4%) treatments should enhance the rate of hydrolysis of these pretreated substrates [51].

## Conclusions

An initial mild steam pretreatment of poplar wood was shown to solubilize and recover much of the labile hemicellulose component, while a range of subsequent pretreatment/extraction stages were shown to vary in their ability to enhance the enzymatic hydrolysis of the cellulose-rich residual fractions. At an equivalent level of severity, higher temperatures at intermediate residence times were more effective in selectively solubilizing hemicellulose in the initial pretreatment stage as higher temperature compromised cellulose recovery. A DES composed of lactic acid and betaine was more selective in extracting lignin from the steam-pretreated substrates that was organosolv and soda/AQ post treatments. Although the milder conditions used in DES extraction seemed to preserve the overall structure of cellulose component, the organosolv- and soda/AQ-posttreated substrates were more readily hydrolysed, likely due to the simultaneous removal of the lignin and disruption of the cellulose structure.

## Abbreviations

SP: steam pretreatment
DES: deep eutectic solvent
AQ: anthraquinone
HPLC: high-performance liquid chromatography
XRD: X-ray diffraction patterns
CrI: crystallinity index
DP: degree of polymerization
CED: cupriethylenediamine
SS: Simon’s staining
WRV: water retention value

## References

[1] Sánchez ÓJ, Cardona CA (2008). Trends in biotechnological production of fuel ethanol from different feedstocks. Bioresour Technol.

[2] Achinivu EC, Howard RM, Li G, Gracz H, Henderson WA (2014). Lignin extraction from biomass with protic ionic liquids. Green Chem.

[3] Bozell JJ, Petersen GR (2010). Technology development for the production of biobased products from biorefinery carbohydrates—the US Department of Energy’s “Top 10” revisited. Green Chem.

[4] Clark JH (2007). Green chemistry for the second generation biorefinery—sustainable chemical manufacturing based on biomass. J Chem Technol Biotechnol.

[5] Li J, Henriksson G, Gellerstedt G (2007). Lignin depolymerization/repolymerization and its critical role for delignification of aspen wood by steam explosion. Bioresour Technol.

[6] Schwiderski M, Kruse A, Grandl R, Dockendorf D (2014). Comparison of the influence of a Lewis acid AlCl3 and a Brønsted acid HCl on the organosolv pulping of beech wood. Green Chem.

[7] Pan X, Kadla JF, Ehara K, Gilkes N, Saddler JN (2006). Organosolv ethanol lignin from hybrid poplar as a radical scavenger: relationship between lignin structure, extraction conditions, and antioxidant activity. J Agric Food Chem.

[8] Verdía P, Brandt A, Hallett JP, Ray MJ, Welton T (2014). Fractionation of lignocellulosic biomass with the ionic liquid 1-butylimidazolium hydrogen sulfate. Green Chem.

[9] Fort DA, Remsing RC, Swatloski RP, Moyna P, Moyna G, Rogers RD (2007). Can ionic liquids dissolve wood? Processing and analysis of lignocellulosic materials with 1-*n*-butyl-3-methylimidazolium chloride. Green Chem.

[10] Nguyen TY, Cai CM, Kumar R, Wyman CE (2015). Co-solvent pretreatment reduces costly enzyme requirements for high sugar and ethanol yields from lignocellulosic biomass. Chemsuschem.

[11] Pinkert A, Goeke DF, Marsh KN, Pang S (2011). Extracting wood lignin without dissolving or degrading cellulose: investigations on the use of food additive-derived ionic liquids. Green Chem.

[12] Lan W, Liu CF, Sun RC (2011). Fractionation of bagasse into cellulose, hemicelluloses, and lignin with ionic liquid treatment followed by alkaline extraction. J Agric Food Chem.

[13] Panagiotopoulos IA, Chandra RP, Saddler JN (2013). A two-stage pretreatment approach to maximise sugar yield and enhance reactive lignin recovery from poplar wood chips. Bioresour Technol.

[14] Bura R, Chandra R, Saddler J (2009). Influence of Xylan on the enzymatic hydrolysis of steam-pretreated corn stover and hybrid poplar. Biotechnol Prog.

[15] Zhao X, Cheng K, Liu D (2009). Organosolv pretreatment of lignocellulosic biomass for enzymatic hydrolysis. Appl Microbiol Biotechnol.

[16] Wildschut J, Smit AT, Reith JH, Huijgen WJJ (2013). Ethanol-based organosolv fractionation of wheat straw for the production of lignin and enzymatically digestible cellulose. Bioresour Technol.

[17] Sannigrahi P, Ragauskas AJ, Miller SJ (2010). Lignin structural modifications resulting from ethanol organosolv treatment of Loblolly pine. Energy Fuels.

[18] Abbott AP, Boothby D, Capper G, Davies DL, Rasheed RK (2004). Deep Eutectic Solvents formed between choline chloride and carboxylic acids: versatile alternatives to ionic liquids. J Am Chem Soc.

[19] Francisco M, van den Bruinhorst A, Kroon MC (2012). New natural and renewable low transition temperature mixtures (LTTMs): screening as solvents for lignocellulosic biomass processing. Green Chem.

[20] Sirviö JA, Visanko M, Liimatainen H (2015). Deep eutectic solvent system based on choline chloride-urea as a pre-treatment for nanofibrillation of wood cellulose. Green Chem.

[21] Venica AD, Chen CL, Gratzl JS (2008). Soda-AQ delignification of poplar wood. Part 1: reaction mechanism and pulp properties. Holzforschung.

[22] Anglès MN, Reguant J, Garcia-Valls R, Salvadó J (2003). Characteristics of lignin obtained from steam-exploded softwood with soda/anthraquinone pulping. Wood Sci Technol.

[23] Mou H, Li B, Fardim P (2014). Pretreatment of corn stover with the modified hydrotropic method to enhance enzymatic hydrolysis. Energy Fuels.

[24] Mou HY, Heikkilä E, Fardim P (2013). Topochemistry of alkaline, alkaline-peroxide and hydrotropic pretreatments of common reed to enhance enzymatic hydrolysis efficiency. Bioresour Technol.

[25] Tan SSY, MacFarlane DR, Upfal J, Edye LA, Doherty WOS, Patti AF (2009). Extraction of lignin from lignocellulose at atmospheric pressure using alkylbenzenesulfonate ionic liquid..

[26] Jin Y, Jameel H, Chang H, Phillips R (2010). Green liquor pretreatment of mixed hardwood for ethanol production in a repurposed kraft pulp mill. J Wood Chem Technol.

[27] Chandra RP, Gourlay K, Kim C-S, Saddler JN (2015). Enhancing hemicellulose recovery and the enzymatic hydrolysis of cellulose by adding lignosulfonates during the two-stage steam pretreatment of poplar. ACS Sustain Chem Eng.

[28] Tian D, Zhang X, Lu C, Yuan G, Zhang W, Zhou Z (2014). Solvent-free synthesis of carboxylate-functionalized cellulose from waste cotton fabrics for the removal of cationic dyes from aqueous solutions. Cellulose.

[29] French AD, Santiago Cintrón M (2013). Cellulose polymorphy, crystallite size, and the Segal crystallinity index. Cellulose.

[30] Xiong R, Zhang X, Tian D, Zhou Z, Lu C (2012). Comparing microcrystalline with spherical nanocrystalline cellulose from waste cotton fabrics. Cellulose.

[31] Hu J, Arantes V, Pribowo A (2014). Substrate factors that influence the synergistic interaction of AA9 and cellulases during the enzymatic hydrolysis of biomass. Energy Environ Sci.

[32] Chandra R, Ewanick S, Hsieh C, Saddler JN (2008). The characterization of pretreated lignocellulosic substrates prior to enzymatic hydrolysis, part 1: a modified Simons’ staining technique. Biotechnol Prog.

[33] Overend RP, Chornet E (1987). Fractionation of lignocellulosics by steam-aqueous pretreatments. Philos Trans R Soc A.

[34] Emmel A, Mathias A, Wypych F, Ramos LP (2003). Fractionation of *Eucalyptus grandis* chips by diluted acid-catalysed steam explosion. Bioresour Technol.

[35] Kumar L, Arantes V, Chandra R, Saddler J (2012). The lignin present in steam pretreated softwood binds enzymes and limits cellulose accessibility. Bioresour Technol.

[36] Alvarez Vasco C, Ma R, Quintero M, Guo M, Geleynse S, Ramasamy KK (2016). Unique low-molecular-weight lignin with high purity extracted from wood by deep eutectic solvents (DES): a source of lignin for valorization. Green Chem.

[37] Lee SH, Doherty TV, Linhardt RJ, Dordick JS (2009). Ionic liquid-mediated selective extraction of lignin from wood leading to enhanced enzymatic cellulose hydrolysis. Biotechnol Bioeng.

[38] Pu Y, Jiang N, Ragauskas AJ (2007). Ionic liquid as a green solvent for lignin. J Wood Chem Technol.

[39] Francisco M, Van Den Bruinhorst A, Kroon MC (2013). Low-transition-temperature mixtures (LTTMs): a new generation of designer solvents. Angew Chem Int Ed.

[40] Sun N, Rahman M, Qin Y, Maxim ML, Rodríguez H, Rogers RD (2009). Complete dissolution and partial delignification of wood in the ionic liquid 1-ethyl-3-methylimidazolium acetate. Green Chem.

[41] Fu D, Mazza G, Tamaki Y (2010). Lignin extraction from straw by ionic liquids and enzymatic hydrolysis of the cellulosic residues. J Agric Food Chem.

[42] McDonough TJ (1993). The chemistry of organosolv delignification. Tappi J.

[43] Gierer J (1986). Chemistry of delignification—part 2: reactions of lignins during bleaching. Wood Sci Technol.

[44] Jin Y, Huang T, Geng W, Yang L (2013). Comparison of sodium carbonate pretreatment for enzymatic hydrolysis of wheat straw stem and leaf to produce fermentable sugars. Bioresour Technol.

[45] Pan X, Xie D, Yu RW, Lam D, Saddler JN (2007). Pretreatment of lodgepole pine killed by mountain pine beetle using the ethanol organosolv process: fractionation and process optimization. Ind Eng Chem Res.

[46] Alvira P, Tomás-Pejó E, Ballesteros M, Negro MJ (2010). Pretreatment technologies for an efficient bioethanol production process based on enzymatic hydrolysis: a review. Bioresour Technol.

[47] Nitsos CK, Matis KA, Triantafyllidis KS (2013). Optimization of hydrothermal pretreatment of lignocellulosic biomass in the bioethanol production process. Chemsuschem.

[48] Engström AC, Ek M, Henriksson G (2006). Improved accessibility and reactivity of dissolving pulp for the viscose process: pretreatment with monocomponent edoglucanase. Biomacromol.

[49] Weise U, Maloney T, Paulapuro H (1996). Quantification of water in different states of interaction with wood pulp fibres. Cellulose.

[50] Szijártó N, Horan E, Zhang J, Puranen T, Siika-aho M, Viikari L (2011). Thermostable endoglucanases in the liquefaction of hydrothermally pretreated wheat straw. Biotechnol Biofuels.

[51] Skovgaard PA, Thygesen LG, Jørgensen H, Cardona M, Tozzi E, McCarthy M, Siika-Aho M, Jeoh T (2014). The role of endoglucanase and endoxylanase in liquefaction of hydrothermally pretreated wheat straw. Biotechnol Prog.

